# Positional Cloning of Zinc Finger Domain Transcription Factor *Zfp69*, a Candidate Gene for Obesity-Associated Diabetes Contributed by Mouse Locus *Nidd/SJL*

**DOI:** 10.1371/journal.pgen.1000541

**Published:** 2009-07-03

**Authors:** Stephan Scherneck, Matthias Nestler, Heike Vogel, Matthias Blüher, Marcel-Dominique Block, Mauricio Berriel Diaz, Stephan Herzig, Nadja Schulz, Marko Teichert, Sina Tischer, Hadi Al-Hasani, Reinhart Kluge, Annette Schürmann, Hans-Georg Joost

**Affiliations:** 1 Department of Pharmacology, German Institute of Human Nutrition Potsdam-Rehbruecke, Nuthetal, Germany; 2 Department of Medicine, University of Leipzig, Leipzig, Germany; 3 Emmy Noether and Marie Curie Research Group Molecular Metabolic Control, DKFZ-ZMBH Alliance, German Cancer Research Center, Heidelberg, Germany; The Wellcome Trust Centre for Human Genetics, University of Oxford, United Kingdom

## Abstract

Polygenic type 2 diabetes in mouse models is associated with obesity and results from a combination of adipogenic and diabetogenic alleles. Here we report the identification of a candidate gene for the diabetogenic effect of a QTL (*Nidd/SJL, Nidd1*) contributed by the SJL, NON, and NZB strains in outcross populations with New Zealand Obese (NZO) mice. A critical interval of distal chromosome 4 (2.1 Mbp) conferring the diabetic phenotype was identified by interval-specific congenic introgression of SJL into diabetes-resistant C57BL/6J, and subsequent reporter cross with NZO. Analysis of the 10 genes in the critical interval by sequencing, qRT–PCR, and RACE–PCR revealed a striking allelic variance of *Zfp69* encoding zinc finger domain transcription factor 69. In NZO and C57BL/6J, a retrotransposon (IAPLTR1a) in intron 3 disrupted the gene by formation of a truncated mRNA that lacked the coding sequence for the KRAB (Krüppel-associated box) and Znf-C2H2 domains of *Zfp69*, whereas the diabetogenic SJL, NON, and NZB alleles generated a normal mRNA. When combined with the B6.V-*Lep^ob^* background, the diabetogenic *Zfp69^SJL^* allele produced hyperglycaemia, reduced gonadal fat, and increased plasma and liver triglycerides. mRNA levels of the human orthologue of *Zfp69*, *ZNF642*, were significantly increased in adipose tissue from patients with type 2 diabetes. We conclude that *Zfp69* is the most likely candidate for the diabetogenic effect of *Nidd/SJL*, and that retrotransposon IAPLTR1a contributes substantially to the genetic heterogeneity of mouse strains. Expression of the transcription factor in adipose tissue may play a role in the pathogenesis of type 2 diabetes.

## Author Summary

Type 2 diabetes in humans as well as in obese mice is caused by a combination of adipogenic and diabetogenic gene variants. We have identified a gene that appears to be involved in the pathogenesis of hyperglycaemia in obese mice: in some mouse strains, the gene *Zfp69* is disrupted by a retroviral transposon (IAPLTR1a), which generates a truncated mRNA. Disruption of the gene was associated with a reduced susceptibility for diabetes, whereas the normal allele enhanced hyperglycaemia in obese mice. *Zfp69* encodes a transcription factor which appears to interfere with lipid storage in adipose tissue, and thereby enhances lipid deposition in liver. In humans with type 2 diabetes, mRNA levels of the human orthologue of *Zfp69* (*ZNF642*) were increased in adipose tissue. Thus, the transcription factor ZFP69/ZNF642 may be involved in the pathogenesis of obesity-associated diabetes.

## Introduction

Type 2 diabetes results from the combination of insulin resistance and inadequate insulin secretion, the former being associated with obesity [1]. The risk of developing type 2 diabetes is to approximately 50% inherited [2]. Recently, numerous associations between single nucleotide polymorphisms and the diabetes risk in humans have been identified and confirmed [3]–[7]. However, little is known as to the functional consequences of these SNPs at the molecular, cellular, and physiological level.

Obese mouse strains carrying the *Lep^ob^* (ob) or the *Lep^db^* (db) mutation have proven to be valuable models for the study of the pathophysiology and genetics of type 2 diabetes [8]. In these strains, the adipogenic mutation is necessary, but not sufficient for the development of severe hyperglycaemia and diabetes [9]. Thus, the diabetic phenotype appeared to be conferred by the background strain, and it was assumed that lean mice may carry diabetogenic and/or diabetes-protecting alleles. Furthermore, quantitative trait loci for obesity and hyperglycaemia were separated in outcross experiments of New Zealand Obese (NZO) mice and lean strains, proving the concept that diabetes is the result of a combination of adipogenic and diabetogenic alleles [10]–[13]. Subsequently, two genes that confer diabetes susceptibility of obese strains have been identified. *Sorcs1* is a gene involved in microvasculature function, and contributes to diabetes in BTBR.V(B6)-*Lep^ob^* mice [14]. A variant of *Lisch-like* was shown to be responsible in part for the diabetogenic effect of the DBA background in mice carrying the adipogenic *db* mutation [15]. *Lisch-like* has been suggested to be involved in the development of insulin-producing cells. Thus, positional cloning of mouse diabetes genes may provide major insights into the pathogenesis of obesity-associated diabetes.

**Figure 1:**
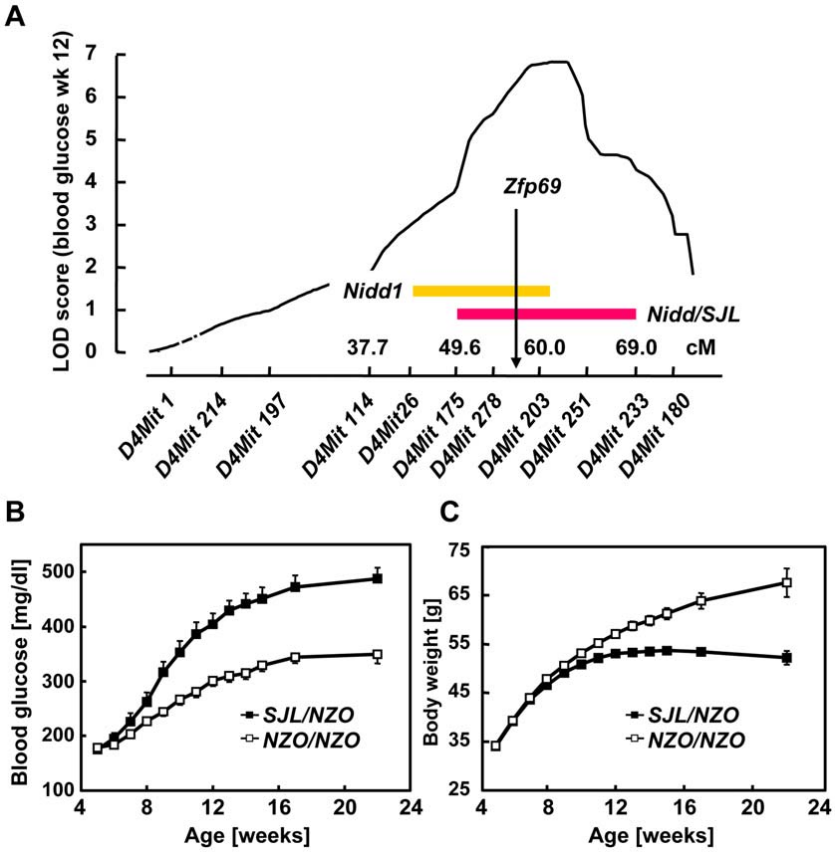
Location and diabetogenic effect of QTL *Nidd/SJL* on distal mouse chromosome 4. (A) LOD score curve of *Nidd/SJL* derived from a (NZOxSJL)N2 backcross population of 207 male mice. The approximate position of the *Nidd1* QTL harboring a diabetogenic allele from NON was obtained from Leiter *et al.* [10]. (B,C) The chromosomal segment *D4Mit175*–*D4Mit233* of SJL was introgressed into B6, and a (NZOxB6.SJL-*Nidd/SJL*)N2 backcross population was generated. Blood glucose (B) and weight gain (C) in male backcross mice were monitored weekly. Data represent means±SE.

We have previously identified a QTL (*Nidd/SJL*) on distal chromosome 4 that aggravated and accelerated diabetes in an outcross population of NZO with the lean SJL strain [11],[12]. This QTL exhibited high LOD scores for the trait blood glucose, and reproducibly doubled the prevalence of diabetes in a NZOxSJL backcross population [12]. In addition, it markedly enhanced the effect of a second diabetes and obesity-modifying gene [16]. The chromosomal position of *Nidd/SJL* is similar to that of a previously described diabetogenic QTL (*Nidd1*, Figure 1) which has been identified in an outcross of NZO with NON [10]. Interestingly, the human syntenic region of *Nidd1* and *Nidd/SJL* (human chromosome 1) comprises a QTL for reduced insulin secretion that was identified in the Pima Indian population [17]. Furthermore, in a recent metaanalysis of diabetogenic mouse QTL, distal chromosome 4 was among the 7 consensus regions with the highest combined LOD scores [18]. Thus, *Nidd/SJL* appeared to be a prime target for positional cloning of a novel mouse diabetes gene.

## Results

### Identification and fine-mapping of a critical diabetogenic interval of *Nidd/SJL*

Figure 1A illustrates the position of the *Nidd/SJL* locus on distal chromosome 4 [11],[12] and its proximity to the previously described *Nidd1* [10]. For further analysis of the QTL, we introgressed a segment of SJL chromosome 4 defined by the markers *D4Mit175* and *D4Mit233* (Figure 1A) into the C57BL/6J (B6) background. These mice (B6.SJL-*Nidd/SJL*) were lean and exhibited no alteration in glucose homeostasis (data not shown). Thus, B6.SJL-*Nidd/SJL* mice were then mated with NZO in order to introduce obesity, and the resulting F1 was intercrossed or backcrossed on NZO. Characterization of the N2 progeny indicated that *Nidd/SJL* carriers exhibited early onsetting hyperglycaemia with blood glucose levels approximately 150 mg/dl higher than in carriers of the NZO allele (Figure 1B), and stopped gaining weight in week 10–12 (Figure 1C). Similar results were obtained in the F2 intercross which showed an additive effect of *Nidd/SJL* (Figure S1). It should be noted that carriers of the NZO allele of *Nidd/SJL* also became hyperglycaemic, although to a much lesser degree than carriers of the SJL allele (Figure 1B), presumably due to other diabetogenic alleles from NZO chromosomes 1 and 15 [10],[11; Vogel *et al.*, unpublished]. These mice, however, continued to gain weight (Figure 1C), indicating that the weight development could be used as an additional criterion to determine the presence or absence of the causal gene in *Nidd/SJL*.

**Figure 2:**
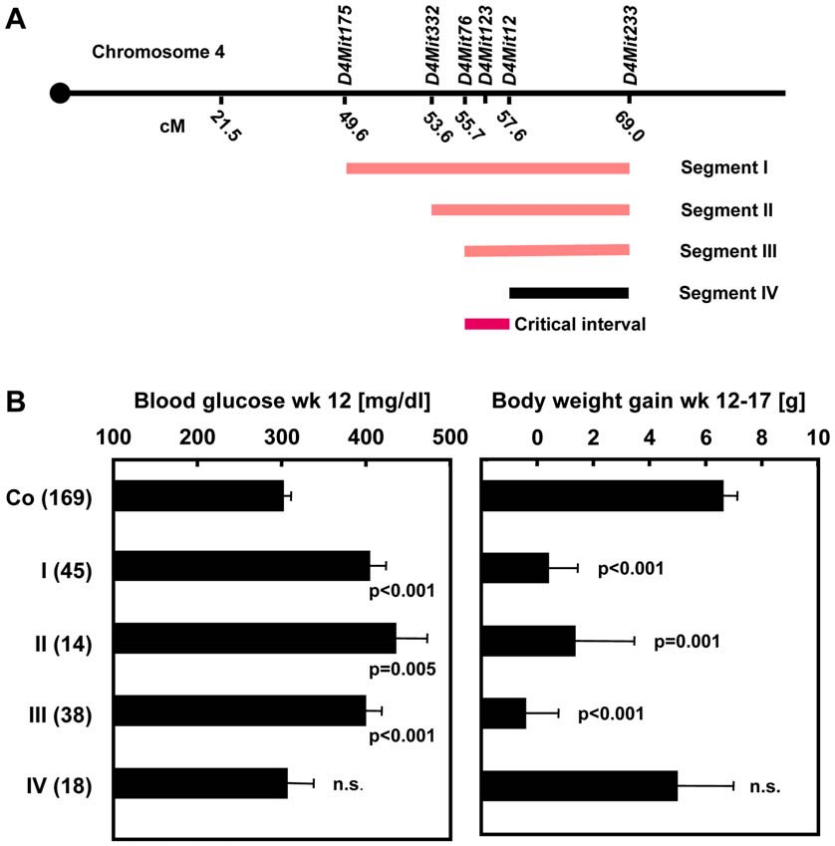
Identification of a critical interval of mouse chromosome 4 harboring the diabetogenic allele *Nidd/SJL*. (A) Map of chromosomal segments I-IV of SJL introgressed into the B6 background. (B) Blood glucose (wk 12) and weight gain (wk 12–17) of male mice from a (NZOxB6.SJL-*Nidd/SJL*)N2 male backcross mice generated with the recombinant congenic lines. Data represent means±SE of a number of mice given in parenthesis.

**Figure 3:**
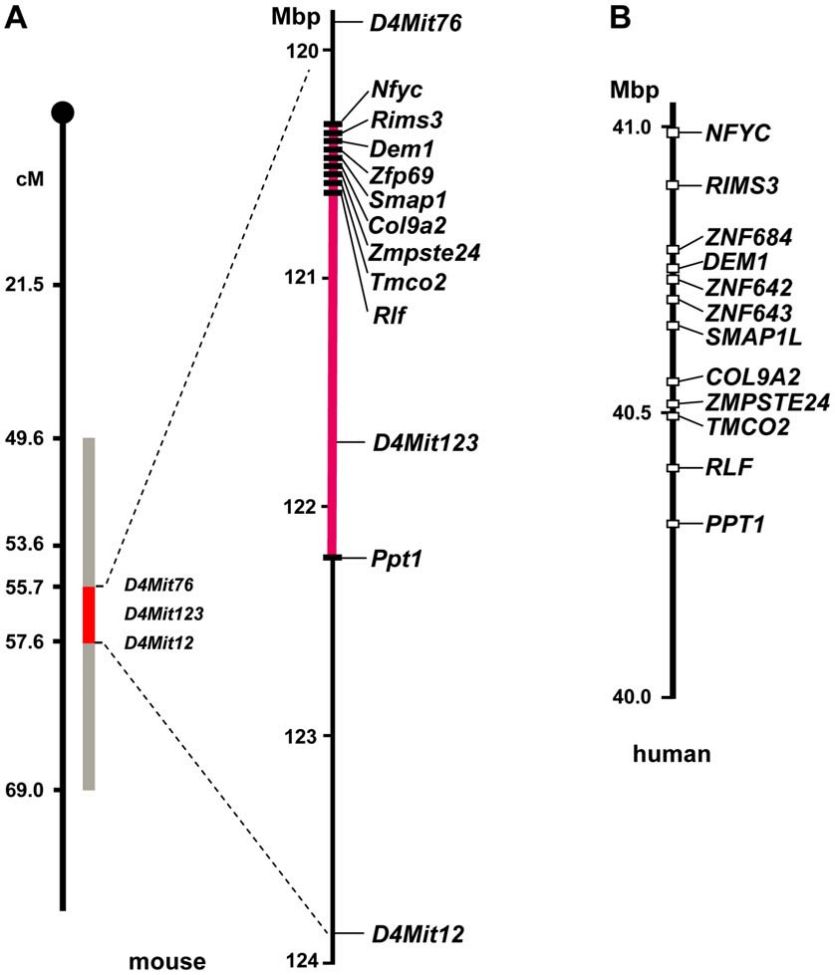
Map of the critical interval of *Nidd/SJL*. (A) The interval was determined by introgression of segments III and IV (Figure 2) of SJL into B6, and was initially defined by the markers *D4Mit76*, *D4Mit123*, and *D4Mit12*. Further fine-mapping with SNPs (Figure S2) restricted the interval to a region between the genes *Nfyc* and *Ppt1* (red segment). (B) Comparison of the segment with the human syntenic region on chromosome 1.

For restriction of the critical segment of *Nidd/SJL*, interval-specific congenic B6.SJL-*Nidd/SJL* mice carrying different segments of the QTL (Figure 2A) were mated with NZO and backcrossed. Characterization of the N2 progeny with regard to their blood glucose levels and development of body weight indicated that segments I, II, and III were diabetogenic (Figure 2B). Segment IV, in contrast, which serendipitously originated from segment III in the final backcross to B6, failed to produce the severe hyperglycaemia and growth arrest. Thus, the critical interval of chromosome 4 comprising the diabetogenic allele was defined by the markers *D4Mit76* and *D4Mit12* (Figure 2A). For further fine mapping we used additional SNPs from the public databases (Figure S2), thereby reducing the critical interval defined by the genotypes of segments III and IV to 2.1 Mbp (Figure 3A and Figure S2). The interval was flanked by *Nfyc* and *Ppt1*, and contained 10 confirmed genes. The human syntenic region contains two additional genes (Figure 3B; *ZNF684 and ZNF643*), presumably duplications of *ZNF642*. Data base searches indicated that these genes are not present in the mouse genome; their closest mouse orthologue is *Zfp69*.

### Analysis of the critical region by sequencing and mRNA profiling

Sequencing of all 10 genes in the critical interval (Figure 3A) identified non-synonymous substitutions in *Zfp69* (T57I, A79V), *Smap2* (T257I), and *Col9A2* (T298I, A482I, R610H). *Zfp69* encodes a transcription factor; the amino acid exchanges are located outside of its functional domains (zinc finger binding domain, KRAB domain). *Smap2* (stromal membrane-associated protein 2; alias *Smap1l*) encodes an ARF-GTPase activating protein which regulates protein trafficking from endosomes to the Golgi [19],[20]; its crystal structure has been determined [21]. In the SJL sequence, threonine 257 is exchanged for isoleucine; the human orthologue also carries an isoleucine in this position. The exchange is classified as ‘tolerated’ by the SIFT program which predicts deleterious amino acid substitutions [22]. *Col9A2* encodes a collagen subunit which is predominantly expressed in cartilage [23]. The three substitutions identified in the SJL sequence are classified as ‘tolerated’ by the SIFT program. Thus, none of the amino acid exchanges in *Smap2* and *Col9a2* are likely candidates explaining the diabetogenic effect of *Nidd/SJL*.

**Figure 4:**
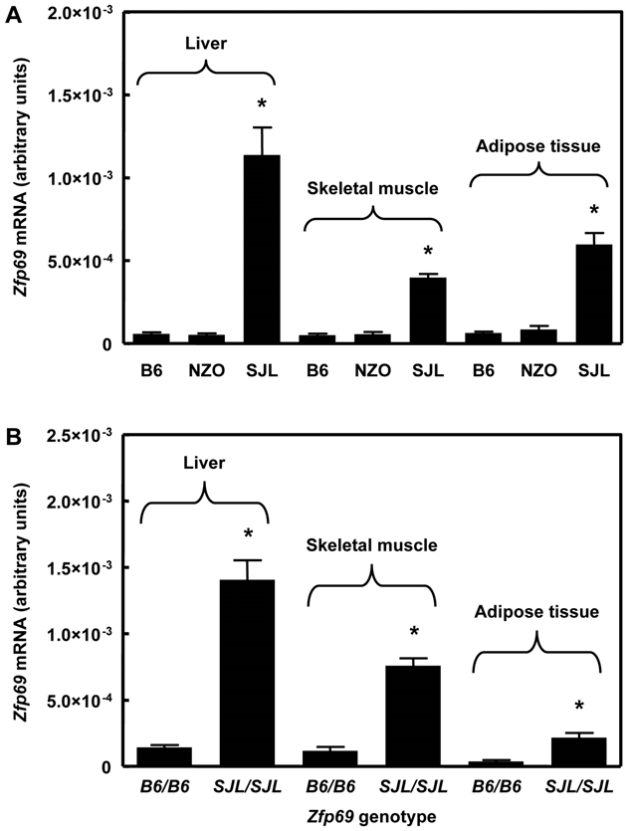
Expression of *Zfp69* is suppressed in NZO and B6. (A) mRNA levels of *Zfp69* in liver, skeletal muscle, and epididymal adipose tissue from parental SJL, B6, and NZO strains as determined by quantitative RT-PCR. (B) Differential expression of *Zfp69* mRNA is dependent on the genotype of the *Zfp69* locus (*D4Mit123*). For this analysis, congenic B6.SJL-*Nidd/SJL* homozygous for the *ob*-mutation were used. Asterisks indicate significance of difference to B6/NZO (p<0.001).

Next, we determined the expression of all 10 confirmed genes in liver, muscle, and adipose tissue of SJL, NZO, and B6 by quantitative PCR (data for adipose tissue shown in Figure S3). mRNA of *Col9a2* and *Tmco2* was undetectable in these tissues. With the exception of *Zfp69*, none of the other investigated genes exhibited significant and consistent differences in their expression. As is illustrated in Figure 4, mRNA levels of *Zfp69* differed markedly between the strains NZO, B6, and SJL: mRNA levels of *Zfp69* were nearly undetectable in NZO and B6, but were present in SJL (Figure 4A). Analysis of tissues from congenic B6.SJL-*Nidd/SJL* mice indicated that the expression of *Zfp69* was dependent on the genotype (B6 or SJL) of the critical interval of *Nidd/SJL* (Figure 4B). These data suggested that an allelic variation of *Zfp69* itself had caused its differential expression.

### Identification of a loss-of-function variant of *Zfp69*

**Figure 5:**
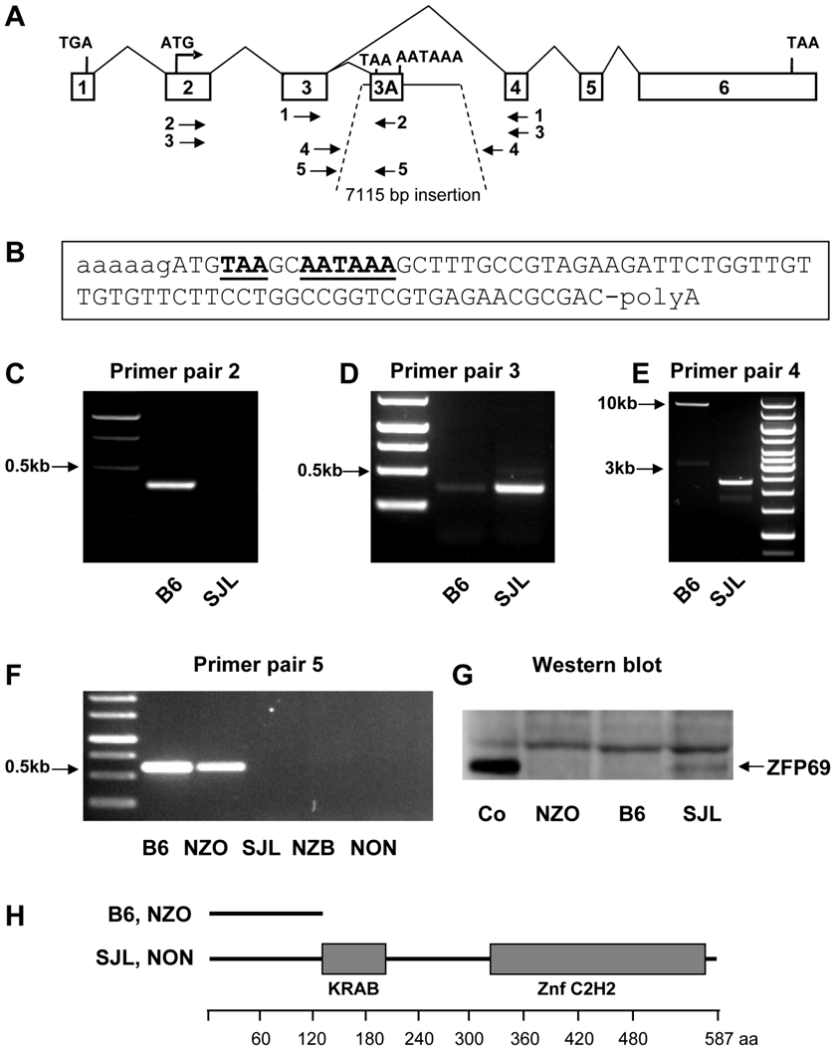
Loss-of-function of *Zfp69* in NZO and B6 by alternative mRNA splicing and intronic polyadenylation. (A) Genomic organisation and PCR primer pairs used for characterization of *Zfp69*. (B) Sequence of the additional exon 3A (capital letters) that was polyadenylated and spliced to exon 3 as identified by 3′-RACE–PCR. (C,D) Identification of the cDNA variants in B6 and SJL by PCR with downstream primers derived from exons 3A and 4. (E) Detection of the 7 kbp insertion in intron 3 of B6. (F) allelic variation of *Zfp69* in different mouse strains with known contribution of distal chromosome 4 to diabesity. (G) Western blot of nuclear extracts isolated liver of NZO, B6, and SJL mice. Co, recombinant ZFP69 generated by transfection of COS-7 cells with *Zfp69* cDNA. (H) Model of the domain structure of ZFP69 and the truncated variant (B6, NZO).

In order to test the possibility that the marked difference in the RT-PCR signal (primer pair 1 in Figure 5A) between NZO and SJL was due to the formation of different mRNA species, we analysed the *Zfp69* cDNA by RACE-PCR. Products of 5′-RACE corresponded with the reference sequence (Accession number ENSMUST00000106280) and were identical in the two strains. By 3′-RACE, however, we detected a shorter cDNA in B6 and NZO that contained only the first three exons fused to a short segment of intron 3 (Figure 5B; alternative exon 3A in Figure 5A); this segment comprised a stop codon and a polyadenylation site. PCR with a primer matching exon 3A indicated that the shorter cDNA was expressed in B6 but not in SJL (Figure 5C). Conversely, the full length cDNA of *Zfp69* comprising exon 4 was nearly undetectable in B6 (Figure 5D), consistent with the results of the quantitative PCR shown in Figure 4. Further characterization of intron 3 by PCR (Figure 5E) and sequencing indicated that B6 and NZO carry an inserted retrotransposon (IAPLTR1a) which functions as a gene trap by causing intronic polyadenylation and alternative splicing. Accordingly, no immunoreactive ZFP69 protein was detected in B6 and NZO with antiserum against a C-terminal peptide (Figure 5G). The retrotransposon was also detected in NZL and BKS which are strains related with NZO and B6, respectively. In contrast, SJL, NZB, NON (Figure 5F), and 5 other strains we tested (Table S3) lacked the 7115 bp insertion.

*Zfp69* is a member of the subfamily of zinc finger transcription factors that comprise a N-terminal KRAB and a zinc finger binding C2H2 domain (Figure 5H, [24],[25]. The shorter mRNA generated in B6 and NZO encodes a truncated protein which can be considered a loss-of-function variant since it lacks both the KRAB and the (DNA binding) C2H2 domain of the transcription factor.

### Allelic variation of *Zfp69* in mouse strains NZO, B6, NZB, SJL, and NON corresponds with expression of *Zfp69* and with the diabetogenic effect of chromosome 4 in three outcross populations

Several outcross experiments generating obese mouse populations have previously been performed that showed the presence (NZOxNON, [10]; NZOxSJL, [11]; NZOxNZB, Schmolz *et al.*, unpublished) or absence (NZOxB6, Vogel *et al.*, unpublished) of a diabetogenic allele in the vicinity of *D4Mit278* on chromosome 4. According to these data, NON and SJL contributed a major diabetogenic effect (*Nidd1* and *Nidd/SJL*). In addition, distal chromosome 4 of NZB (*D4Mit203*) contributed to the hyperglycaemia of the (NZOxNZB)F2 (blood glucose in wk 22: genotype NZO/NZO, 301±32; NZO/NZB, 389±19; NZB/NZB, 376±28 mg/dl; p<0.05 for differences to NZO/NZO). Based on these data, we expected that SJL, NON, and NZB carry an identical (diabetogenic) allele of *Zfp69*, and that NZO and B6 both carry a diabetes-suppressing allele. Indeed, the contribution of the different mouse strains to hyperglycaemia in the intercross populations corresponded with the allelic variation of *Zfp69* in these strains (Figure 5F). These data are consistent with the hypothesis that loss of function of *Zfp69* supresses diabetes, and that complementation by the ‘wild-type’ allele as in SJL enhances obesity-associated diabetes.

### Insertion of the retrotransposon IAPLTR1a produces aberrant mRNA species of eight genes in the B6 genome

Endogenous retroviral elements such as IAP and ETn/MusD retrotransposons have previously been shown to be significant genomic mutagens [26], and appear to contribute substantially to the genetic heterogeneity of mouse strains. In order to test the possibility that IAPLTR1a insertion generates variant transcripts of other genes in the B6 genome, we used a bioinformatic approach and identified all insertions of the retrotransposon by an alignment with its 338 bp LTR sequence. This alignment identified 33 integrations into introns of genes. Subsequently, all EST clones that mapped to the position of these genes were identified and aligned with the reference cDNAs. With this procedure, a total of 8 genes including the previously reported *Adamts13* [27] were found (Table 1) that generated aberrant mRNA species (premature polyadenylation or alternative transcription start) due to the insertion of the IAPLTR1a.

**Table 1:** Gene variants in B6 mice caused by integration of the IAPLTR1a_Mm sequence.

Gene	Position	Aberrant EST/mRNA	Modification
*Fmo1*	1qH2.1	AI132203	truncated mRNA
		AK042457	
		BB242793	
		BF121776	
*Adamts13*	2qA3	AB071302	truncated mRNA
		EU034706	
*Cdk5rap1*	2qH1	AW908483	alternative transcription start
		BB842254	
		DV051174	
		W82224	
		W82241	
*Sgip1*	4qC6	AK049616	truncated mRNA
*Zfp69*	4qD2.2	BB795296	truncated mRNA
		CJ244124	
		CJ244263	
		CJ244311	
*Grid2*	6qC1	BY035709	truncated mRNA
		BY036240	
*Cpne8*	15qE3	AI853527	truncated mRNA
		AK005311	
		AW548480	
		BC048551	
		BC076564	
		BQ553434	
		BQ553435	
		BU846828	
		BY705490	
		CA451877	
		DV040130	
		DV054241	
*Lrrc33*	16qB2	BY035709	alternative transcription start
		BY036240	

BLAT search with 338 bp of the IAPLTR1a_Mm sequence identified 202 integrations into the B6 genome (90 retrotransposons flanked with LTR sequences and 22 isolated LTR sequences). Thirty-three of the integrations were located in introns of genes. In 8 transcripts modifications caused by the LTR motive were identified; 6 led to a truncated mRNA, and 2 generated an alternative transcription start. Variants in the *Adamts13* mRNA caused by the IAPLTR1a_Mm sequence were previously described [27].

### Characterization of B6-*ob/ob* mice carrying the diabetogenic *Zfp69* allele

**Figure 6:**
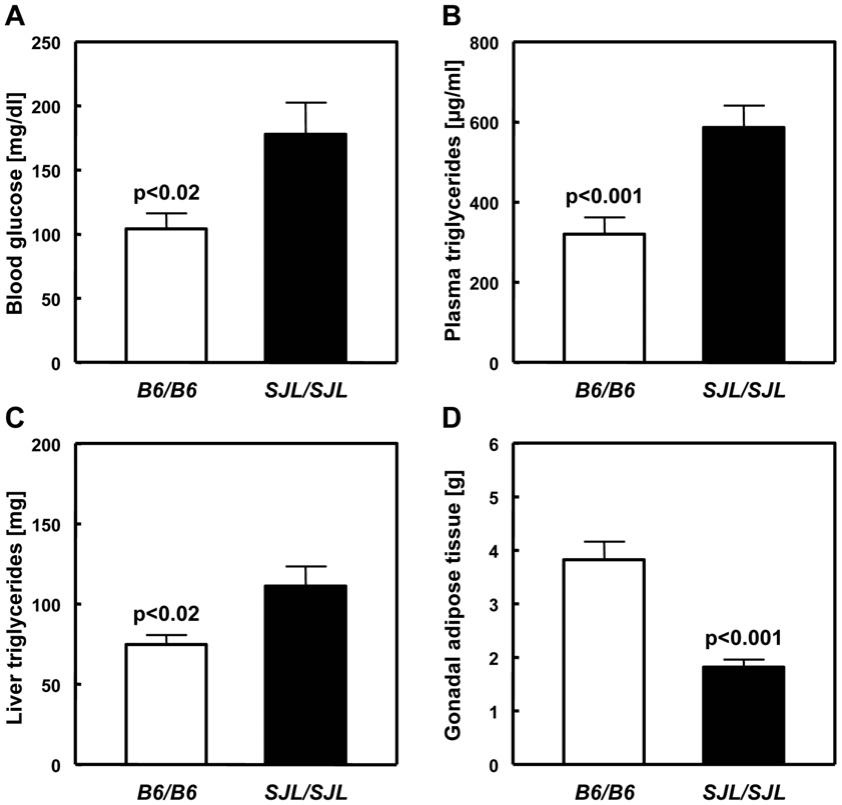
Effect of the *Zfp69^SJL^* allele in B6.V-*Lep^ob^* mice. *Nidd/SJL* was combined with a B6.V-*Lep^ob^* background by intercross, and male B6-*ob/ob*.SJL-*Nidd/SJL* (*SJL/SJL*) were compared with obese control mice (*B6/B6*). Postabsorptive (6 h fasting) blood glucose levels (A), liver triglyceride content (C), and weight of gonadal fat pads (D) were determined at week 13, plasma triglycerides (B) at week 28. Data represent means±SE of 10 (A,C,D) or 24 mice (B).

In order to study the functional consequences of the presence or absence of *Zfp69* mRNA as seen in B6 *vs*. SJL, we combined the *ob* mutation with *Nidd/SJL* on the B6 background. Obese mice homozygous for the SJL allele of *Zfp69* exhibited the same time course of weight gain as carriers of the B6 allele (loss-of-function variant; Figure S4A), but significantly higher blood glucose (Figure 6A and Figure S4B) and plasma triglyceride levels (Figure 6B). Most strikingly, the SJL genotype of 13 weeks old B6-*ob/ob*.SJL-*Nidd/SJL* mice was associated with a significant increase (30%) in liver triglyceride content (Figure 6C) and weight (data not shown), and with a pronounced reduction (60%) of gonadal (epididymal) fat (Figure 6D). The reduction in gonadal fat mass appeared to precede the hepatosteatosis of B6-*ob/ob*.SJL-*Nidd/SJL* mice, since in younger animals (8 weeks), a difference between genotypes in gonadal fat, but not in hepatic weight which paralleles hepatosteatosis was observed (Figure S5). Thus, *Nidd/SJL* might have caused a moderate lipid storage defect, and a redistribution of triglycerides to ectopic stores.

### Interaction of the diabetogenic alleles *Nidd/SJL* (*Zfp69^SJL^*) and *Nob1* (*Tbc1d1^NZO^*)

We have previously reported that the diabetogenic effect of *Nidd/SJL* was accelerated and aggravated by a QTL on chromosome 5 (*Nob1*) [12]. More recently, we have identified the RabGAP *Tbc1d1* as the gene responsible for the effect of *Nob1* [16]. *Tbc1d1^NZO^* reduced fatty acid oxidation in muscle, thereby enhancing obesity and diabetes susceptibility. Increased levels of ectopic triglycerides caused by *Zfp69* would therefore explain the interaction of *Nidd/SJL* with *Nob1*. In order to strengthen this point, we analysed the data of the NZOxSJL intercross for an earlier time point (Figure S6). This analysis indicated that the diabetogenic *Zfp69^SJL^* allele required *Tbc1d1^NZO^* in order to significantly increase blood glucose and plasma insulin levels in week 10.

### Increased mRNA levels of *ZNF642*, the human orthologue of *Zfp69*, in white adipose tissue of patients with type 2 diabetes

**Figure 7:**
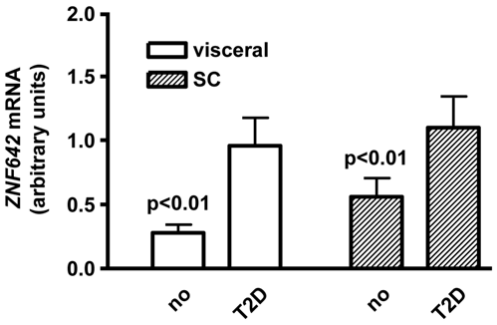
Expression of *ZNF642* in adipose tissue of human individuals with (T2D) and without (no) type 2 diabetes. mRNA levels were determined by qRT–PCR in subcutaneous (sc) and visceral adipose tissue from 67 controls and 31 individuals with type 2 diabetes.

In order to test the possibility that the human orthologue of *Zfp69* is involved in the pathogenesis of human type 2 diabetes, we determined its expression in omental and subcutaneous white adipose tissue of diabetic and control individuals. As is illustrated in Figure 7, mRNA levels of *ZNF642* were significantly higher in diabetic patients than in controls in both omental and subcutaneous adipose tissue. In addition, there was a significant correlation of HbA1c levels with *ZNF642* mRNA (r = 0.32; p<0.006). Subgroup analysis indicated that the correlation was significant in overweight (BMI>25; r = 0.34; p = 0.002) but not in lean (BMI<25; r = −0.09; p = 0.74) individuals.

## Discussion

The present data identify the zinc finger domain transcription factor *Zfp69* as the most likely candidate for the diabetogenic effect of the mouse QTL *Nidd1* and *Nidd/SJL* which aggravates and accelerates obesity-associated diabetes in the NZO strain, and enhances hyperglycaemia in B6-*ob/ob* mice. The following arguments can be made in favour of this conclusion:

1. With interval-specific congenics, we defined a critical genomic interval with 10 genes that was required to enhance diabetes in NZO mice,
2. of the 10 genes located in that interval, *Zfp69* exhibited the most pronounced allelic variation in that the gene was ‘trapped’ by a retrotransposon,
3. this allelic variation corresponded with the diabetogenic or diabetes-resistant effect of the QTL in five mouse strains, and
4. expression of the human orthologue of *Zfp69* is increased in adipose tissue of human diabetics, supporting the hypothesis that the gene is involved in adipose tissue function.

Surprisingly, diabetes appears to be produced by ‘rescue’ of a loss-of-function variant of *Zfp69*: NZO mice and the diabetes-resistant B6 strain express a truncated mRNA, whereas the diabetogenic allele from SJL and NON produces a ‘normal’ expression of *Zfp69*.

Identification of *Zfp69* as the causal gene crucially depends on exclusion of other variations in the critical region. Firstly, *Zfp69* was the only gene in the region exhibiting a significant differential expression in liver, adipose tissue, muscle or pancreas. Secondly, the T257I substitution in SMAP2 is outside of the functional domains of the ARF-GTPase activating protein [19],[20],[21], corresponds with the human sequence, and is classified as ‘tolerated’ by a method predicting deleterious substitutions [22]. Thirdly, the three non-synonymous exchanges in *Col9a2* were also classified as ‘tolerated’. Furthermore, we failed to detect mRNA of *Col9a2* in insulin-sensitive tissues or pancreas by PCR. The gene encodes a subunit of the extracellular matrix protein collagen type IX which is involved in cartilage and bone function [23]. Loss-of-function mutations cause multiple epiphyseal dysplasia in humans, and skeletal abnormalities in mice [28],[29]. Thus, none of the other nine genes in the critical region are likely candidates for the diabetogenic effect of *Nidd/SJL*.

In order to further elucidate the diabetogenic effect of *Nidd/SJL*, and to link it with a cellular function of *Zfp69^SJL^*, we have studied B6.SJL-*Nidd/SJL* mice rendered obese by the *ob* mutation (B6.V-*Lep^ob^* X B6.SJL-*Nidd/SJL*). Because of their monogenic obesity, these mice are much more homogeneous than the polygenic (NZOxSJL)F2 intercross population. The effects observed in these mice are consistent with the hypothesis that *Nidd/SJL* produced a redistribution of triglycerides from gonadal adipose tissue to ectopic stores such as liver, thereby causing hyperglycaemia through an aggravated insulin resistance. With such a scenario, we hypothesize that *Zfp69^SJL^* primarily causes a reduced storage capacity of epididymal adipose tissue. *Zfp69^SJL^* belongs to a family of transcription factors that comprise the conserved Krüppel-associated box (KRAB) in addition to the zinc finger DNA binding domain [25]. The KRAB domain appears to activate co-repressors, resulting in a suppression of target genes [30]. By analogy, we speculate that the normal *Zfp69* allele suppresses genes required for expansion of adipose tissue stores. It should be noted, however, that this hypothesis requires definitive proof by a direct identification of the genes regulated by *Zfp69*.

Interestingly, the above described scenario would explain the previously observed interaction between the diabetogenic *Nidd/SJL* allele and *Nob1/Tbc1d1* [12]. We have recently shown that the normal *Tbc1d1* allele reduces fatty acid oxidation in muscle, thereby enhancing obesity and diabetes susceptibility [16]. Redistribution of triglycerides caused by *Zfp69* would enhance the deleterious effects of the reduced fat oxidation, and explain the accelerated onset of diabetes observed in the presence of both diabetogenic alleles [12]. However, we cannot fully rule out additional effects of *Zfp69* on other tissues such as muscle or pancreas.

It should be noted that the diabetogenic effect of the *Zfp69* variant is markedly dependent on interaction with other genes contributed by the background strain. *Zfp69* requires obesity in order to produce hyperglycaemia (‘diabesity’), and needs other diabetogenic alleles in order to produce beta cell failure, hypoinsulinaemia, and weight loss. So far, we could not detect beta cell destruction on the B6 background. On the NZO background, it was the interaction with NZO alleles on chromosomes 1 and 15 that enhanced the diabetogenic effect of *Zfp69* [Vogel *et al.*, unpublished].

The production of an aberrant mRNA by alternative splicing is common in human inherited disease. Here, we have identified an unusual mechanism: a truncated *Zfp69* mRNA was generated by insertion of a retrotransposon comprising a polyadenylation signal and a splicing acceptor site into intron 3. By a similar mechanism, expression of the endothelin B receptor is reduced in piebald mice [31]. Furthermore, it was shown recently that human soluble VEGF receptor is generated, and its abundance regulated, by intronic polyadenylation and alternative splicing [32]. In addition, an IAP retrotransposon causes intronic polyadenylation of the mouse *Adamts13* gene [27]. Our *in-silico* search identified at least 7 additional genes with insertions of the IAPLTR1a that produced aberrant transcripts by the same mechanism of intronic polyadenylation and alternative splicing. Together with our functional data on the trapping of *Zfp69* by IAPLTR1a, this finding supports the previous suggestion [26] that insertion of retroviral elements is an important contributor to the genetic heterogeneity of mouse strains.

## Materials and Methods

### Animals

NZO mice from our own colony (NZO/HIBomDife: Dr. R. Kluge, German Institute of Human Nutrition, Nuthetal, Germany), SJL (SJL/NBom, Taconic, M+B, Ry, Denmark), and B6 (C57BL/6JCrl, Charles River, Sulzfeld, Germany) were used throughout. Mice were housed at a temperature of 22°C with a 12:12 hours light-dark cycle (lights on at 6:00 a.m.) in type II or type III macrolon cages with soft wood bedding. Standard chow (maintenance diet for rats and mice, No. V153xR/M-H, Ssniff, Soest, Germany) contained (w/w) 19% protein, 3.3% fat, and 54.1% carbohydrates, with 23%, 8%, and 69% of total digestible energy (11.8 kJ/g) from protein, fat, and carbohydrates, respectively. The high-fat diet (No. C1057, Altromin, Lage, Germany) contained (w/w) 17% protein, 15% fat, and 47% carbohydrates, with 17%, 35%, and 48% of total digestible energy (16.2 kJ/g) from protein, fat, and carbohydrates. The animals were kept in accordance with the NIH guidelines for care and use of laboratory animals, and all experiments were approved by the Ethics Committee of the Ministry of Agriculture, Nutrition and Forestry of the State of Brandenburg, Germany.

### Breeding strategy

SJL mice were backcrossed three times to B6. The progeny was genotyped with microsatellite markers and selected for the genotype of *Nidd/SJL*. The different interval-specific congenic B6.SJL-*Nidd/SJL* mice were mated with NZO. The resulting F1 generation was backcrossed to NZO (N2) or intercrossed (F2). B6-*ob/ob*.SJL-*Nidd/SJL* mice were generated by mating B6.SJL-*Nidd/SJL* animals (defined by markers D4Mit175 and D4Mit251) with B6.V-*Lep^ob^* mice heterozygous for the *ob* allele. Residual SJL donor DNA from other chromosomes as determined by genome-wide SNP genotyping (KBioscience, UK) was 2.9% in the (NZOxB6.SJL-*Nidd/SJL*)N2 progeny (Figure 2), and 7.7% in B6-*ob/ob*.SJL-*Nidd/SJL* mice (Figure 6, Figure S4, S5, S6). For linkage analysis and phenotypic characterization, male mice were used throughout.

### Analysis of body composition

Body fat and lean mass were determined with a nuclear magnetic resonance spectrometer (Bruker Minispec instrument, Echo Medical Systems, Houston, TX, USA). Conscious mice were placed in an applied static field for 0.9 minutes [33]. In addition, body weights were measured with an electronic scale.

### Serum parameters

Blood samples were collected at 8:00–9:00 a.m. from mice that had free access to food and water unless indicated otherwise. Glucose levels were determined with a glucometer elite (Bayer HealthCare, Leverkusen, Germany). Triglyceride levels were measured with Triglyceride Reagent (Sigma, Steinheim, Germany) according to manufacturers' instructions. Values were corrected for free glycerol using Free Glycerol Reagent (Sigma).

### Hepatic triglycerides

Hepatic triglyceride content was determined by an enzymatic assay (Randox, Crumlin, United Kingdom) after chloroform/methanol extraction according to manufacturers' instructions.

### Genotyping

DNA was prepared from mouse tails with a DNA isolation kit based on a salt precipitation method (InViTek, Berlin, Germany). Animals were genotyped for polymorphic microsatellite markers (Table S1) by PCR with oligonucleotide primers obtained from MWG (Ebersberg, Germany), and microsatellite length was determined by non-denaturing polyacrylamide gel electrophoresis. Genotyping of SNPs was performed by sequencing.

### Sequencing

Sequencing of DNA was performed with a 3130*xl* Genetic Analyzer (Applied Biosystems, Darmstadt, Germany) using the BigDye Terminator v3.1 Cycle Sequencing Kit (Applied Biosystems). Sequence analysis was done by SeqScape software 2.5 (Applied Biosystems).

### Quantitative real-time PCR

Total RNA from epididymal mouse white adipose tissue was isolated with the RNeasy Lipid Tissue Mini Kit (QIAGEN, Hilden, Germany) according to manufacturers' instructions. Total RNA from liver and skeletal muscle was extracted with peqGOLD RNA Pure reagent (PeqLab Biotechnologie GmbH, Erlangen, Germany). First strand cDNA synthesis was prepared with 2.0 µg total RNA, random hexamer primer, and SuperscriptIII reverse transcriptase (Invitrogen, Carlsbad, CA). Quantitative real-time PCR was performed with an Applied Biosystems 7300 Real-time PCR system, with TaqMan Gene Expression Master Mix (Applied Biosystems), 25 ng cDNA, and TaqMan Gene Expression Assays (Applied Biosystems, Table S2).

### RACE PCR

Rapid amplification of cDNA ends was performed with the FirstChoice RLM-RACE Kit (Ambion, Darmstadt, Germany) according to manufacturers' instructions.

### Nuclear extract preparation and western blot analysis

Nuclear extracts were prepared from livers of NZO, SJL, and C57BL/6 mice as described previously [34] and analyzed by western blotting [35] with an affinity purified polyclonal antibody raised against a Zfp69-specific peptide (KRQEGNKLENPESS).

### Analysis of human ZNF642 mRNA expression in subcutaneous and visceral adipose tissue

Paired samples of subcutaneous and visceral adipose tissue were obtained from 98 Caucasian men and women, 31 individuals with type 2 diabetes and 67 with normal glucose tolerance test, who underwent open abdominal surgery for weight reduction surgery, cholecystectomy, abdominal injuries, or explorative laparotomy. 19 individuals were lean as defined by a BMI<25 kg/m^2^ and 79 subjects were overweight or obese (BMI>25 kg/m^2^). Samples were immediately frozen in liquid nitrogen after sampling. All subjects gave written informed consent before taking part in the study which was approved by the ethics committee of the University of Leipzig.

Total RNA was isolated from adipose tissue samples with TRIzol (Life Technologies, Grand Island, NY), and 1 µg RNA was reverse transcribed with standard reagents (Life Technologies). Human *ZNF642* gene expression was measured by quantitative real-time RT-PCR in a fluorescent temperature cycler by TaqMan assay. From each RT-PCR, 2 µl cDNA as well as 1 µl of primer/probe mixture (MWG) was amplified in a 20 µl PCR with the Universal Master Mix Reagent from Applied Biosystems according to the manufacturers' instructions. Samples were incubated in an ABI PRISM 7000 sequence detector (Applied Biosystems) for an initial denaturation at 95°C for 10 min, followed by 40 PCR cycles, each cycle consisting of 95°C for 15 s, 60°C for 1 min, and 72°C for 1 min. The following primers were used: human *ZNF642*; left primer: CAT GGA TGG CAG AGA AAG AAG; right primer: GCT CCT GTG AAA TGG TAC TC; dual-labeled probe: CCA GGA GAT CCC AGT TCA GAC TTG A. The 18sRNA served as endogenous control and was determined by a premixed assay on demand for human *18S rRNA* (ABI). Human *ZNF642* mRNA expression was calculated using the Delta CT method [36].

### Statistical analysis

Means of body weights, blood glucose, and insulin levels of the backcross (Figure 2) and intercross progeny (Figure S1, Figure S6) were compared by ANOVA (post-hoc tests: Dunnett's or Games-Howell test, depending on the homogeneity of variances) after testing for homogeneity of variances by Levene's test. Blood glucose values were log-transformed before the analysis. Differences between *B6/B6* and *SJL/SJL* genotypes (Figure 6, Figure S5) were tested by two-tailed Student's t-test. Expression levels determined by quantitative real-time PCR were compared by the nonparametric Kruskal-Wallis H-test (Figure 4, Figure S3).

## Supporting Information

Figure S1 Diabetic hyperglycaemia and diabetes-associated growth retardation in male (NZOxB6.SJL-*Nidd/SJL*)F2 mice carrying the SJL allele of *Nidd/SJL* on the NZO background. After weaning, mice were kept on a high-fat diet (15% (w/w) fat, 47% carbohydrates, 17% protein), and blood glucose and body weight were monitored weekly. Only mice carrying the complete *Nidd/SJL* locus or the corresponding NZO allele were included in the experiment. (A) Time course of non-fasted blood glucose. Data represent means±SE of 34, 70, and 110 homozygous (for SJL allele), heterozygous, and control mice, respectively. (B) Time course of body weight gain. Means±SE of the same number of animals as in A.
(0.19 MB TIF)

Figure S2 Localisation of SNPs and microsatellite markers used for fine mapping of the critical interval as defined by the backcross animals N2-III and N2-IV carrying the segments III and IV. Yellow colour depicts heterozygosity for the SJL allele. The critical interval is highlighted by the red frame.
(0.44 MB TIF)

Figure S3 Relative expression of genes located in the critical interval of *Nidd/SJL*. mRNA levels in epididymal adipose tissue were determined by quantitative RT-PCR, and data were normalized for values obtained from B6. mRNA of *Col9a2* and *Tmco2* was not detectable after 35 PCR cycles. Data are means±SD of 5 mice in each group.
(0.12 MB TIF)

Figure S4 Weight gain and postabsorptive blood glucose in B6.V-*Lep^ob^* mice with or without the *Zfp69^SJL^* allele. After weaning, male homozygous B6-*ob/ob*.SJL-*Nidd/SJL* (*SJL/SJL*) mice and obese controls (*B6/B6*) were kept on a high-fat diet, and body weight (A) and 6 h fasting blood glucose (B) was monitored weekly. (A) Data represent means±SE of 26, 36, and 21 homozygous (*SJL/SJL*), heterozygous, and control mice (*B6/B6*), respectively. (B) Data represent means±SE of 14 homozygous (*SJL*/*SJL*) and 15 control mice (*B6/B6*).
(0.18 MB TIF)

Figure S5 Fat depots and liver triglycerides in B6.V-*Lep^ob^* mice with or without the *Zfp69^SJL^* allele. Liver weights and weights of gonadal (epididymal), subcutaneous, and mesenteric adipose tissue were determined in 8 weeks old homozygous B6-*ob/ob*.SJL-*Nidd/SJL* (*SJL/SJL*) and obese control mice (*B6/B6*). Data represent means±SE of 6 (*SJL/SJL*) and 8 (*B6/B6*) mice.
(0.12 MB TIF)

Figure S6 Interaction of the variant *Zfp69* and *Tbc1d1* alleles in a backcross of NZO with SJL. Blood glucose (A) and immunoreactive insulin (B) was determined in 10 weeks old male (NZOxSJL)N2 progeny (N = 207) that were stratified according to the indicated genotype. The SJL allele of *Tbc1d1* represents a loss-of-function variant and enhances fatty acid oxidation in muscle (16); the SJL allele of *Zfp69* reduces fat storage in gonadal adipose tissue (Figure 6C).
(0.20 MB TIF)

Table S1 Microsatellite markers for genotyping of the *Nidd/SJL* locus.
(0.04 MB DOC)

Table S2 TaqMan Gene Expression Assays (Applied Biosystems).
(0.04 MB DOC)

Table S3 Presence (+) or absence (−) of the IAPLTR1a retrotransposon in the *Zfp69* gene of different mouse strains.
(0.04 MB DOC)
