# Supplementary figures and images for: Positional Cloning of Zinc Finger Domain Transcription Factor Zfp69, a Candidate Gene for Obesity-Associated Diabetes Contributed by Mouse Locus Nidd/SJL
Source: PLoS Genet. 2009 Jul 3;5(7):e1000541. doi: 10.1371/journal.pgen.1000541 (PMC2696593; doi:10.1371/journal.pgen.1000541)

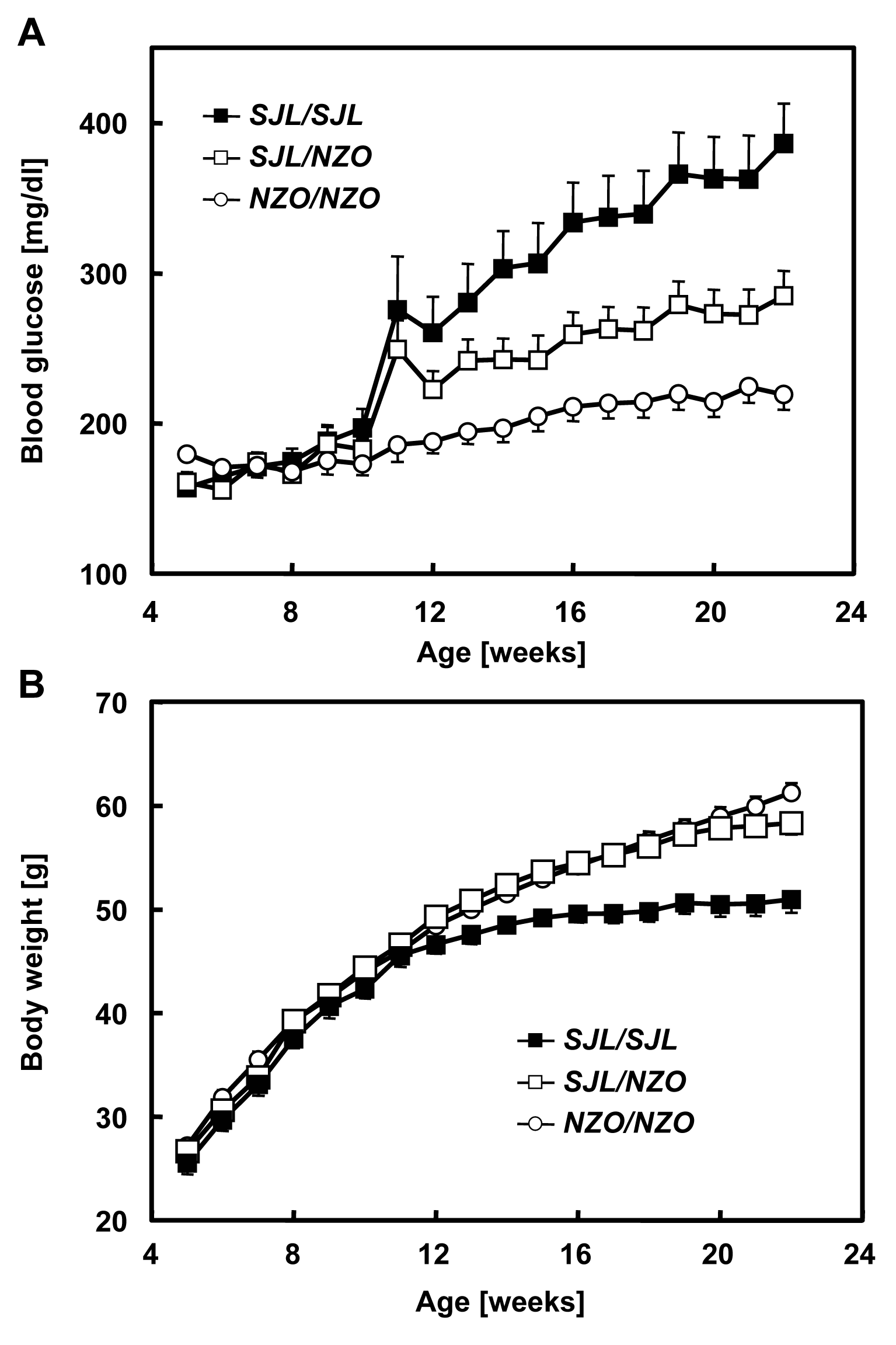

Supplement: Figure S1 — Diabetic hyperglycaemia and diabetes-associated growth retardation in male (NZOxB6.SJL-Nidd/SJL)F2 mice carrying the SJL allele of Nidd/SJL on the NZO background. After weaning, mice were kept on a high-fat diet (15% (w/w) fat, 47% carbohydrates, 17% protein), and blood glucose and body weight were monitored weekly. Only mice carrying the complete Nidd/SJL locus or the corresponding NZO allele were included in the experiment. (A) Time course of non-fasted blood glucose. Data represent means±SE of 34, 70, and 110 homozygous (for SJL allele), heterozygous, and control mice, respectively. (B) Time course of body weight gain. Means±SE of the same number of animals as in A. (0.19 MB TIF) [file pgen.1000541.s001.tif]

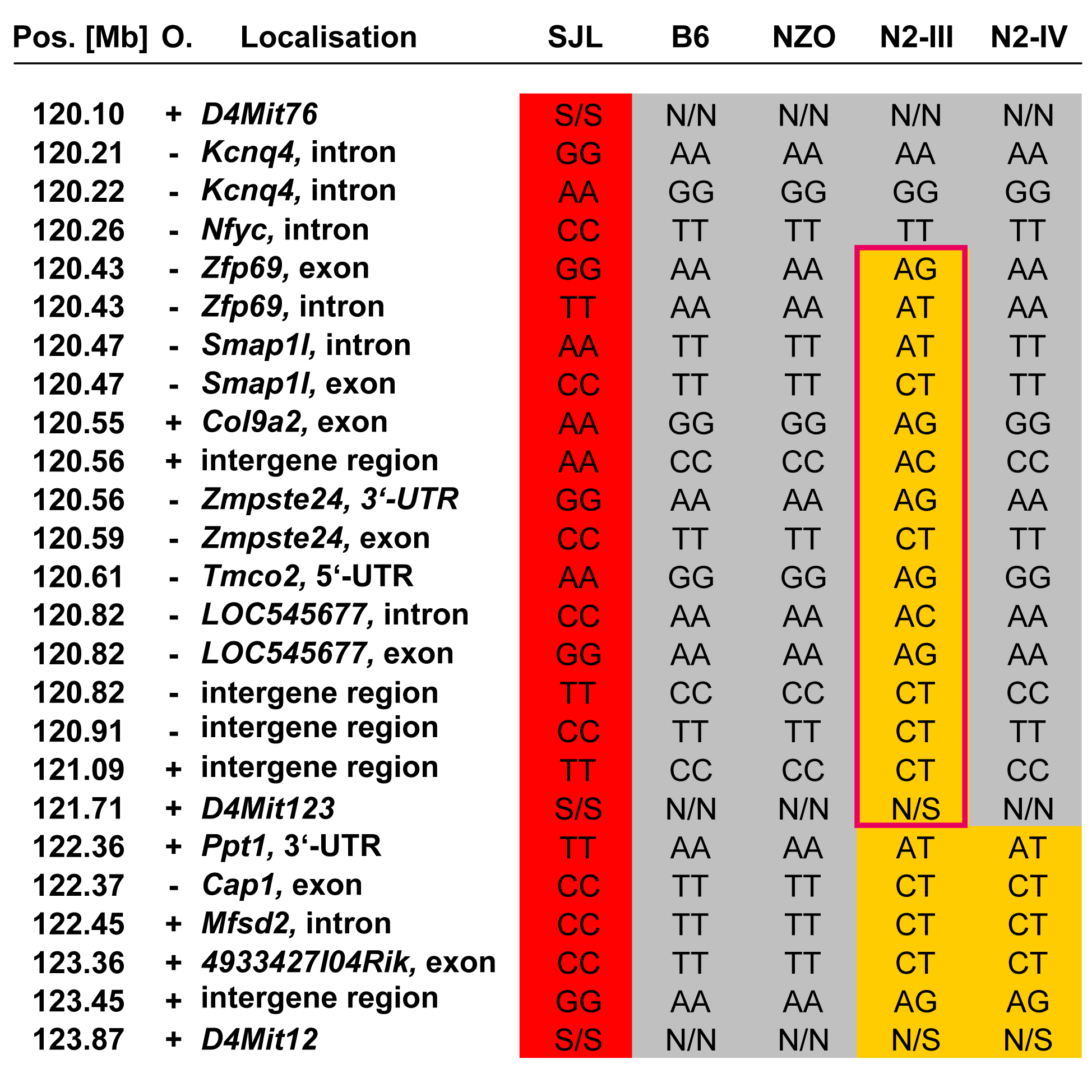

Supplement: Figure S2 — Localisation of SNPs and microsatellite markers used for fine mapping of the critical interval as defined by the backcross animals N2-III and N2-IV carrying the segments III and IV. Yellow colour depicts heterozygosity for the SJL allele. The critical interval is highlighted by the red frame. (0.44 MB TIF) [file pgen.1000541.s002.tif]

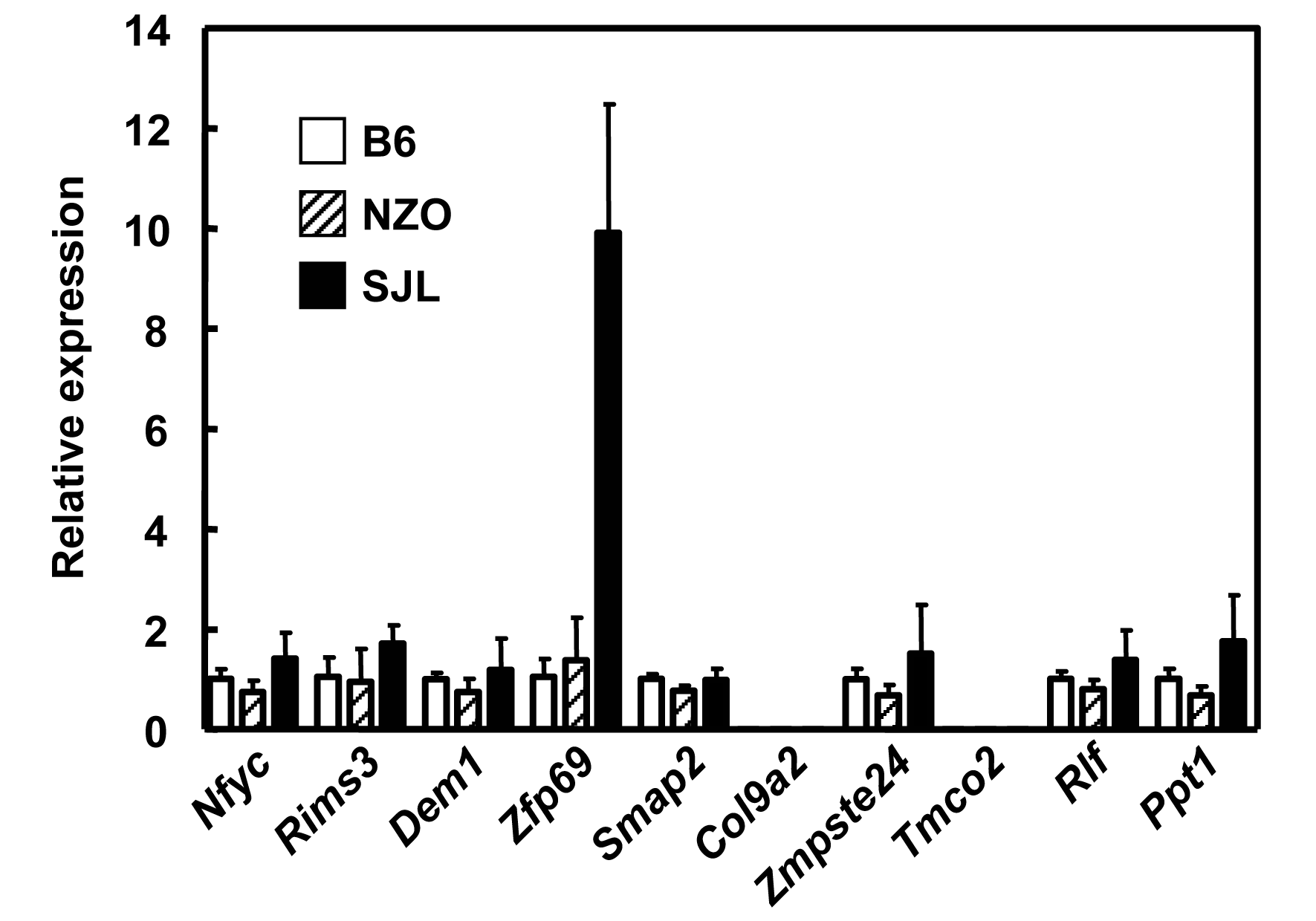

Supplement: Figure S3 — Relative expression of genes located in the critical interval of Nidd/SJL. mRNA levels in epididymal adipose tissue were determined by quantitative RT-PCR, and data were normalized for values obtained from B6. mRNA of Col9a2 and Tmco2 was not detectable after 35 PCR cycles. Data are means±SD of 5 mice in each group. (0.12 MB TIF) [file pgen.1000541.s003.tif]

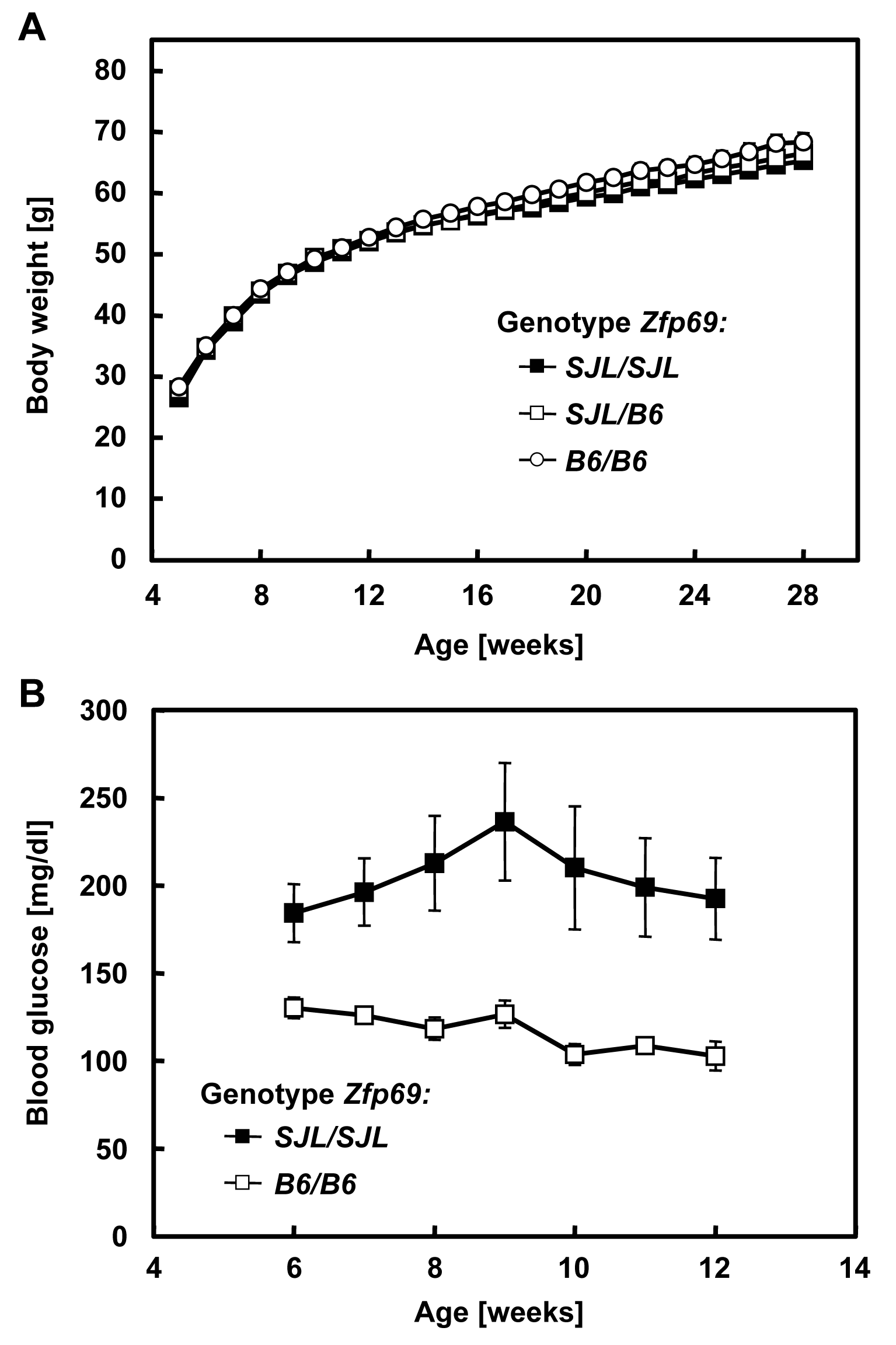

Supplement: Figure S4 — Weight gain and postabsorptive blood glucose in B6.V-Lepob mice with or without the Zfp69SJL allele. After weaning, male homozygous B6-ob/ob.SJL-Nidd/SJL (SJL/SJL) mice and obese controls (B6/B6) were kept on a high-fat diet, and body weight (A) and 6 h fasting blood glucose (B) was monitored weekly. (A) Data represent means±SE of 26, 36, and 21 homozygous (SJL/SJL), heterozygous, and control mice (B6/B6), respectively. (B) Data represent means±SE of 14 homozygous (SJL/SJL) and 15 control mice (B6/B6). (0.18 MB TIF) [file pgen.1000541.s004.tif]

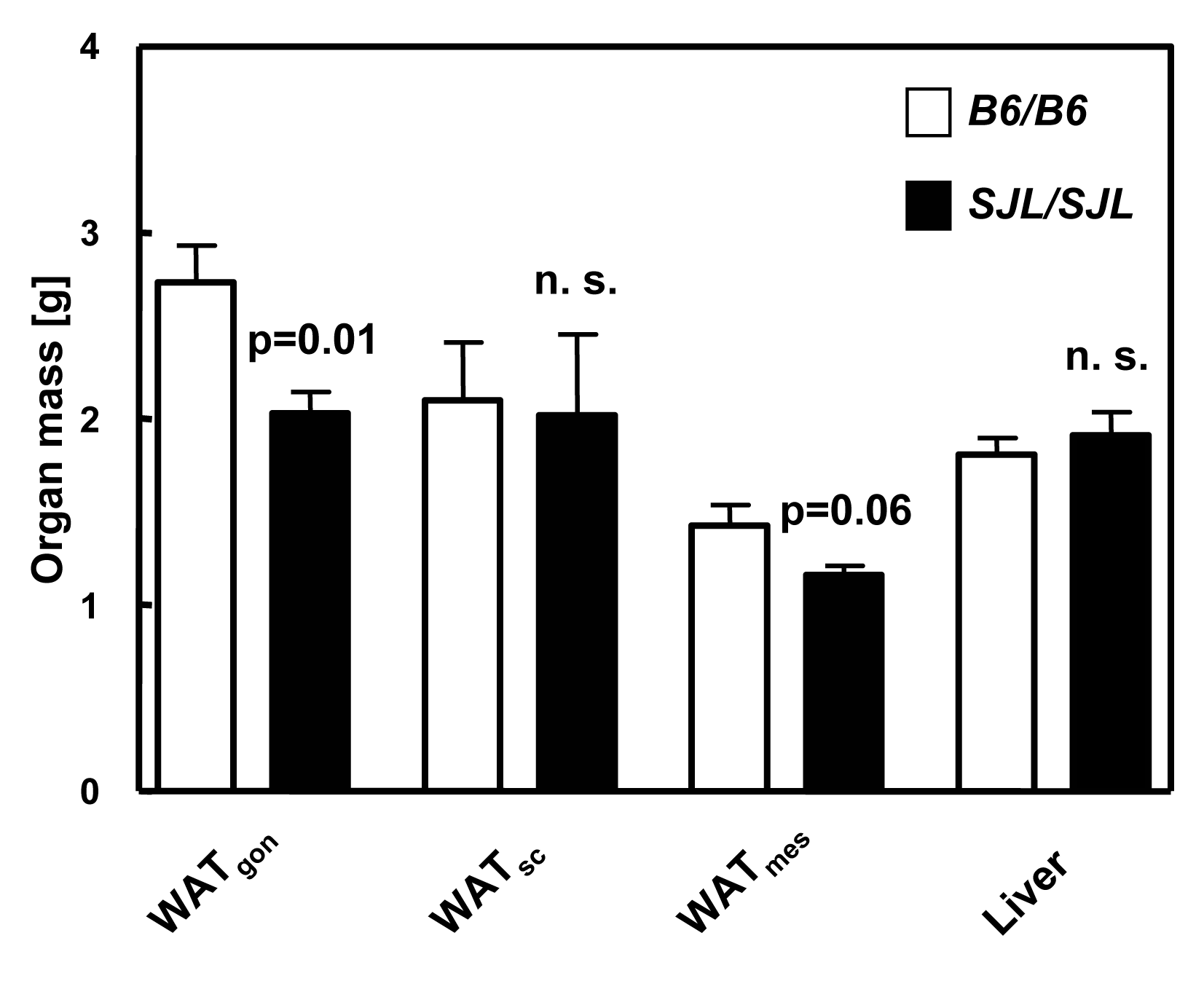

Supplement: Figure S5 — Fat depots and liver triglycerides in B6.V-Lepob mice with or without the Zfp69SJL allele. Liver weights and weights of gonadal (epididymal), subcutaneous, and mesenteric adipose tissue were determined in 8 weeks old homozygous B6-ob/ob.SJL-Nidd/SJL (SJL/SJL) and obese control mice (B6/B6). Data represent means±SE of 6 (SJL/SJL) and 8 (B6/B6) mice. (0.12 MB TIF) [file pgen.1000541.s005.tif]

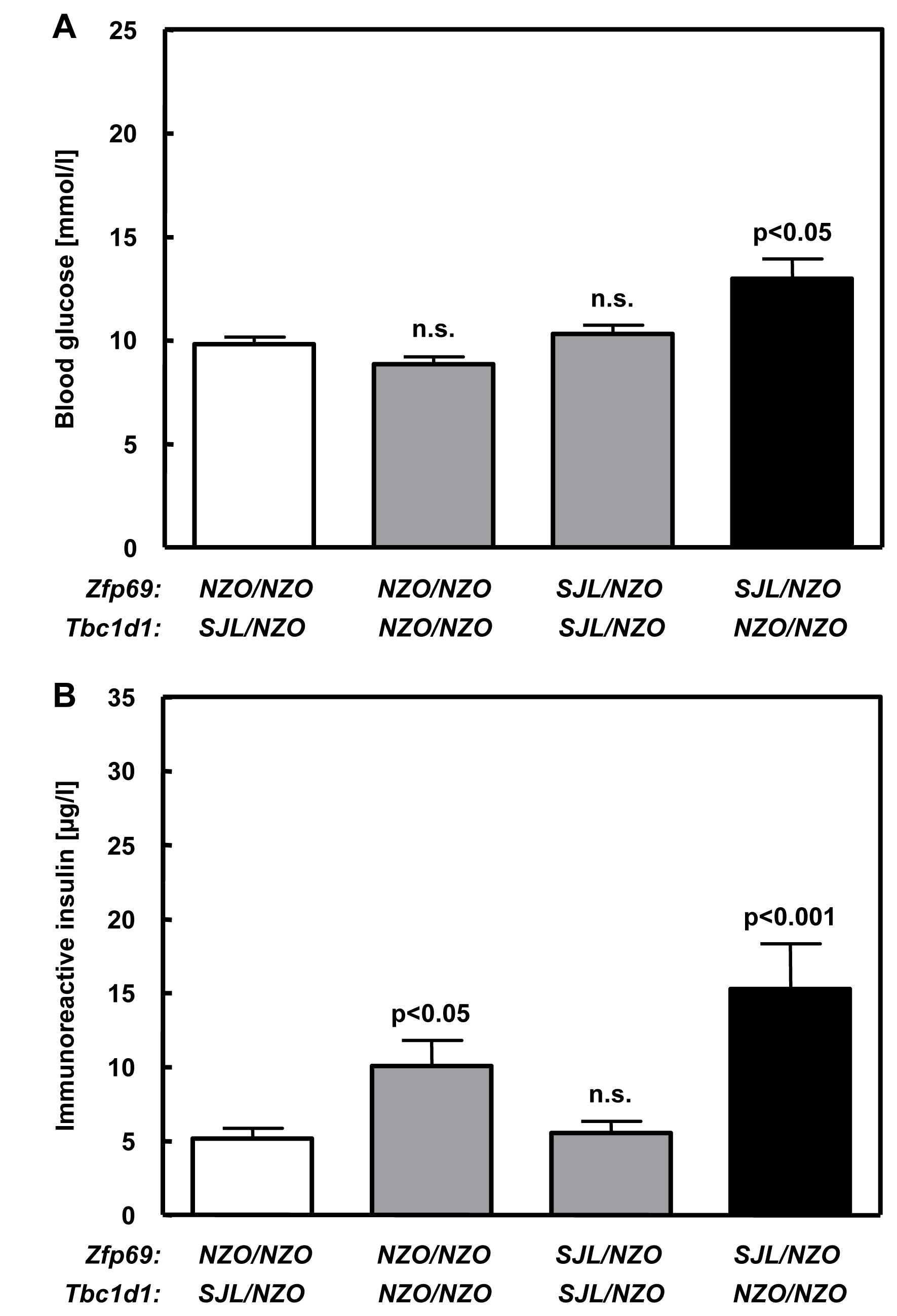

Supplement: Figure S6 — Interaction of the variant Zfp69 and Tbc1d1 alleles in a backcross of NZO with SJL. Blood glucose (A) and immunoreactive insulin (B) was determined in 10 weeks old male (NZOxSJL)N2 progeny (N = 207) that were stratified according to the indicated genotype. The SJL allele of Tbc1d1 represents a loss-of-function variant and enhances fatty acid oxidation in muscle (16); the SJL allele of Zfp69 reduces fat storage in gonadal adipose tissue (Figure 6C). (0.20 MB TIF) [file pgen.1000541.s006.tif]
