# Supplementary material for: Positional Cloning of Zinc Finger Domain Transcription Factor Zfp69, a Candidate Gene for Obesity-Associated Diabetes Contributed by Mouse Locus Nidd/SJL
Source: PLoS Genet. 2009 Jul 3;5(7):e1000541. doi: 10.1371/journal.pgen.1000541 (PMC2696593; doi:10.1371/journal.pgen.1000541)
Supplement: Table S1 — Microsatellite markers for genotyping of the Nidd/SJL locus. (0.04 MB DOC) [file pgen.1000541.s007.doc]

Supplementary Table 1: Microsatellite markers for genotyping of the *Nidd/SJL* locus

| **Marker** | **Position [bp]** | **Position [cM]** | **5´-*Primer*** | **3´-*Primer*** |
| --- | --- | --- | --- | --- |
| *D4Mit175* | 99897429-99897539 | 49.60 | CCT GAC ACA GCC CAC TCC | CTC CCC AGA TAT GCC TTG AA |
| *D4Mit31* | 106610362-106610482 | 51.30 | ACG AGT TGT CCT CTG ATC AAC A | AGC CAG AGC AAA CAC CAA CT |
| *D4Mit199* | 111372990-111373189 | 53.60 | CTA CCA TGG TCT CAT AAA TTG CC | TTA GAT GGC AAG AGT AAG ACA AAC A |
| *D4Mit332* | 113989926-113990074 | 53.60 | TCA ATC CCA TTG GCT ATA TAT GC | TGA GAA ACC TCT CCA GCA CC |
| *D4Mit76* | 120102698-120102879 | 55.70 | TGA AGG AAC CTG AAG CAA GG | ACC TCC CAG GAG TGT CCA G |
| *D4Mit123** | 121713230-121713374 | 57.40 | TCC TTT ATT CTC CTA GAC ACC ACA | AAC ATG TAT GCA AGT TCA ATC TCC |
| *D4Mit123** | 121974862-121975012 | 57.40 | TCC TTT ATT CTC CTA GAC ACC ACA | AAC ATG TAT GCA AGT TCA ATC TCC |
| *D4Mit12* | 123873467-123873663 | 57.60 | GCT TGC TTT AGG AGT GTG CC | TAT TTG CTC TCC ATT TCC CC |
| *D4Mit338* | 124842714-124842814 | 59.50 | CTG ACA AAG TAA GTC AAG GTC AAT G | TGA TTA TGT GCT TCA GTC CTG G |
| *D4Mit336* | 126864067-126864188 | 59.00 | TTC ATA TAT GTG TAC CAT GGC ATG | CAG GAA CCT ACA TAG GTG AGA GG |
| *D4Mit203* | 129074322-129074426 | 60.00 | GAA TTC TTC CTG GGC CTT TC | CAA GAG CCC AGG TGT GGT AT |
| *D4Mit251* | 136199272-136199385 | 66.00 | AAA AAT CGT TCT TTG ACT TCT ACA TG | TTT AAA AGG GTT TCT TTA TCC TGT G |
| *D4Mit233* | 144491631-144491805 | 69.00 | TGG TCA TGT GTG TCC ATG C | ACT TCA TGT AGC CAG GTG GG |

* *D4Mit123* maps to two positions.
