# Supplementary material for: Positional Cloning of Zinc Finger Domain Transcription Factor Zfp69, a Candidate Gene for Obesity-Associated Diabetes Contributed by Mouse Locus Nidd/SJL
Source: PLoS Genet. 2009 Jul 3;5(7):e1000541. doi: 10.1371/journal.pgen.1000541 (PMC2696593; doi:10.1371/journal.pgen.1000541)
Supplement: Table S2 — TaqMan Gene Expression Assays (Applied Biosystems). (0.04 MB DOC) [file pgen.1000541.s008.doc]

Supplementary Table 2: TaqMan Gene Expression Assays (Applied Biosystems)

| **Gene** | **Assay-ID** | **Reference sequence** | **E-E-boundary** | **Amplicon [bp]** |
| --- | --- | --- | --- | --- |
| *Nfyc* | Mm00711396_m1 | NM_008692.3 | 5-6 | 143 |
| *Rims3* | Mm00805396_m1 | NM_182929.2 | 2-3 | 58 |
| *Dem1* | Mm00546887_m1 | NM_028457.1 | 1-2 | 65 |
| *Zfp69* | Mm01185460_m1 | ENSMUST00000106280 | 3-4 | 82 |
| *Smap2* | Mm00505126_s1 | NM_133716.2 | 10-10 | 126 |
| *Col9a2* | Mm00483872_m1 | NM_007741.1 | 16-17 | 105 |
| *Zmpste24* | Mm00554619_m1 | NM_172700.2 | 1-2 | 65 |
| *Tmco2* | Mm01192446_m1 | NM_001081312.1 | 1-2 | 142 |
| *Rlf* | Mm01181427_m1 | AK162649.1 | 6-7 | 107 |
| *Ppt1* | Mm00477078_m1 | NM_008917.2 | 5-6 | 94 |
| *Actb* | Mm00607939_s1 | NM_007393.1 | 6-6 | 115 |
