# Supplementary material for: Positional Cloning of Zinc Finger Domain Transcription Factor Zfp69, a Candidate Gene for Obesity-Associated Diabetes Contributed by Mouse Locus Nidd/SJL
Source: PLoS Genet. 2009 Jul 3;5(7):e1000541. doi: 10.1371/journal.pgen.1000541 (PMC2696593; doi:10.1371/journal.pgen.1000541)
Supplement: Table S3 — Presence (+) or absence (−) of the IAPLTR1a retrotransposon in the Zfp69 gene of different mouse strains. (0.04 MB DOC) [file pgen.1000541.s009.doc]

Supplementary Table 3: Presence (+) or absence (-) of the IAPLTR1a retrotransposon in the *Zfp69* gene of different mouse strains

| **Mouse strain** | **IAPLTR1a** |
| --- | --- |
| 129S2 | - |
| AKR/J | - |
| BKS.Cg-*m* +/+ *Lepr^db^* | + |
| C57BL/6JCrl | + |
| FVBNHsd | - |
| NON/J | - |
| NZB/Hsd | - |
| NZL/LJ | + |
| NZO/HIBomDife | + |
| Rj:NMRI | - |
| SJL/J | - |
| SJL/NBom | - |
| SM/J | - |
